# Supplementary material for: β-arrestin1 and 2 exhibit distinct phosphorylation-dependent conformations when coupling to the same GPCR in living cells
Source: Nat Commun. 2022 Sep 26;13:5638. doi: 10.1038/s41467-022-33307-8 (PMC9512828; doi:10.1038/s41467-022-33307-8)
Supplement: Supplementary file 1 — Supplementary Information [file 41467_2022_33307_MOESM1_ESM.pdf]

β-arrestin1 and 2 exhibit distinct phosphorylation-dependent conformations when coupling to the same GPCR in living cells

Raphael S. Haider*, Edda S. F. Matthees*, Julia Drube, Mona Reichel, Ulrike Zabel, Asuka Inoue, Andy Chevigné, Cornelius Krasel, Xavier Deupi, Carsten Hoffmann

* Authors contributed equally

To whom correspondence should be addressed: carsten.hoffmann@med.uni-jena.de

Supplementary Information

This .pdf includes:

Supplementary Figures 1-11

Supplementary Tables 1-5

Supplementary References

Supplementary Figure 1

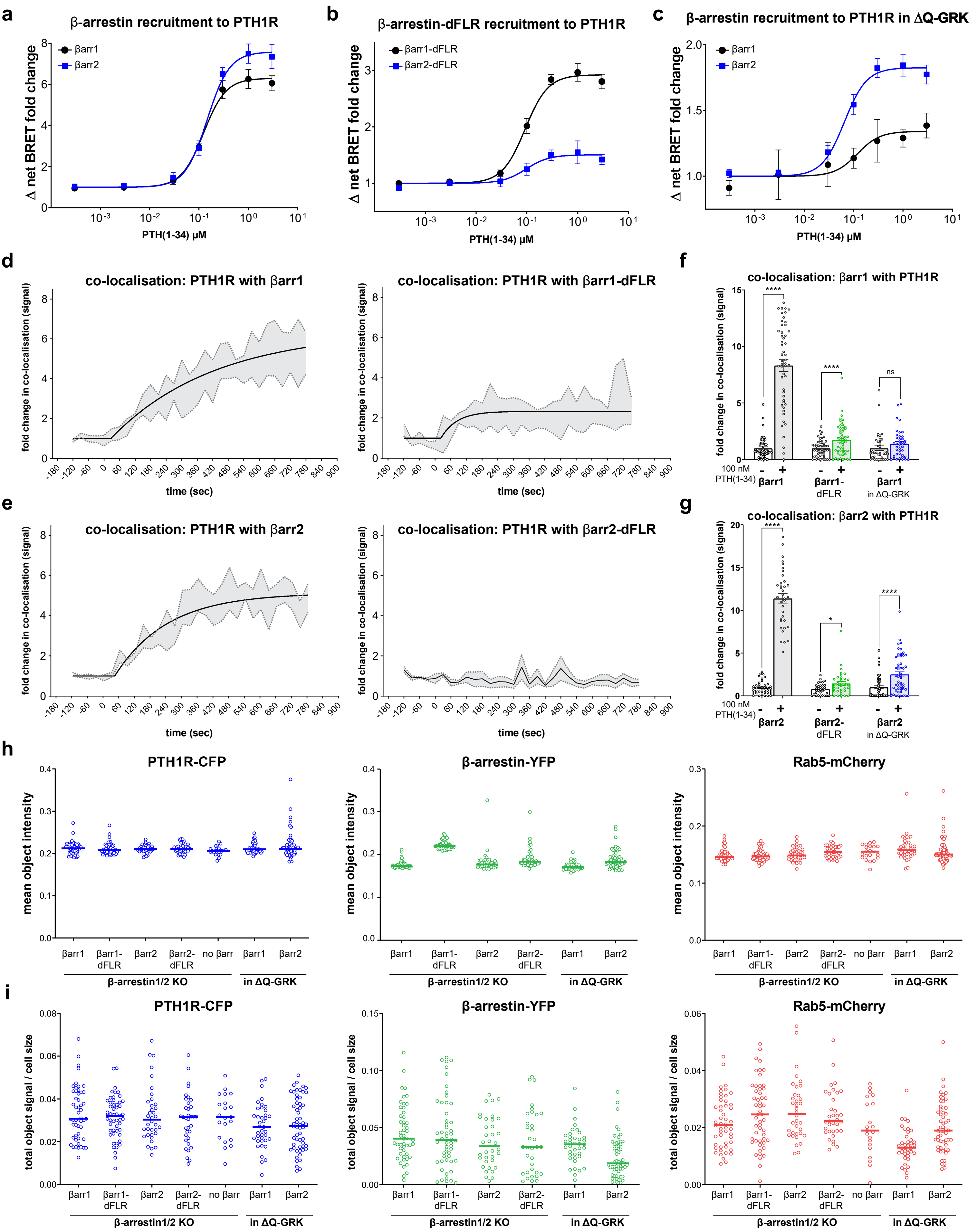

Supplementary Figure 1: a-c Concentration response curves of NanoBRET β-arrestin1 and 2 recruitment to the PTH1R with β-arrestin-WT and -dFLR constructs and in ΔQ-GRK cells from Figure 1b are shown again, sorted into the different conditions and with appropriate axis limits to enable a detailed comparison. d, e Live-cell confocal microscopy of β-arrestin1/2 double knockout cells transfected with PTH1R-CFP, the early endosome marker Rab5-mCherry and β-arrestin1-YFP WT or dFLR constructs (d) or β-arrestin2-YFP WT or dFLR constructs (e). Four representative movies of stimulation with 100 nM PTH(1-34) for 15 minutes were used for the quantification of co-localisation between PTH1R-CFP and the respective β-arrestin1 and 2-YFP constructs over time using Squassh and SquasshAnalyst, represented as mean fold change in co-localisation signal ± SEM. f, g Analogous quantification calculated using Squassh and SquasshAnalyst (number of images per respective condition; βarr1 (39), βarr1-dFLR (43), βarr2 (33), β arr2-dFLR (27)), represented as mean fold change in co-localisation signal + SEM, of paired images, acquired before and after stimulation with 100 nM PTH(1-34) for 15 minutes. Representative images are shown in Figure 1c, e. The statistical significance between paired images, acquired before and after stimulation was calculated by two-sided paired t test (*, $p<0.05$; **, $p<0.01$; ***, $p<0.001$; ****, $p<0.0001$) with exact p-values as follows: βarr1 and βarr1-dFLR $p<0.0001$, βarr1 in ΔQ-GRK $p=0.1791$, βarr2 and βarr2 in ΔQ-GRK $p<0.0001$, βarr2-dFLR $p=0.0134$. h and i show specific segmentation parameters (mean object intensity (h) and total object signal per cell size (i)) for all basal images (number of images per condition; βarr1 (39), βarr1-dFLR (43), no βarr (17), βarr1 in ΔQ-GRK (38), βarr2 (33), βarr2-dFLR (27), βarr2 in ΔQ-GRK (50)) that were subjected to further image analysis.

Source data is provided as a source data file.

Supplementary Figure 2

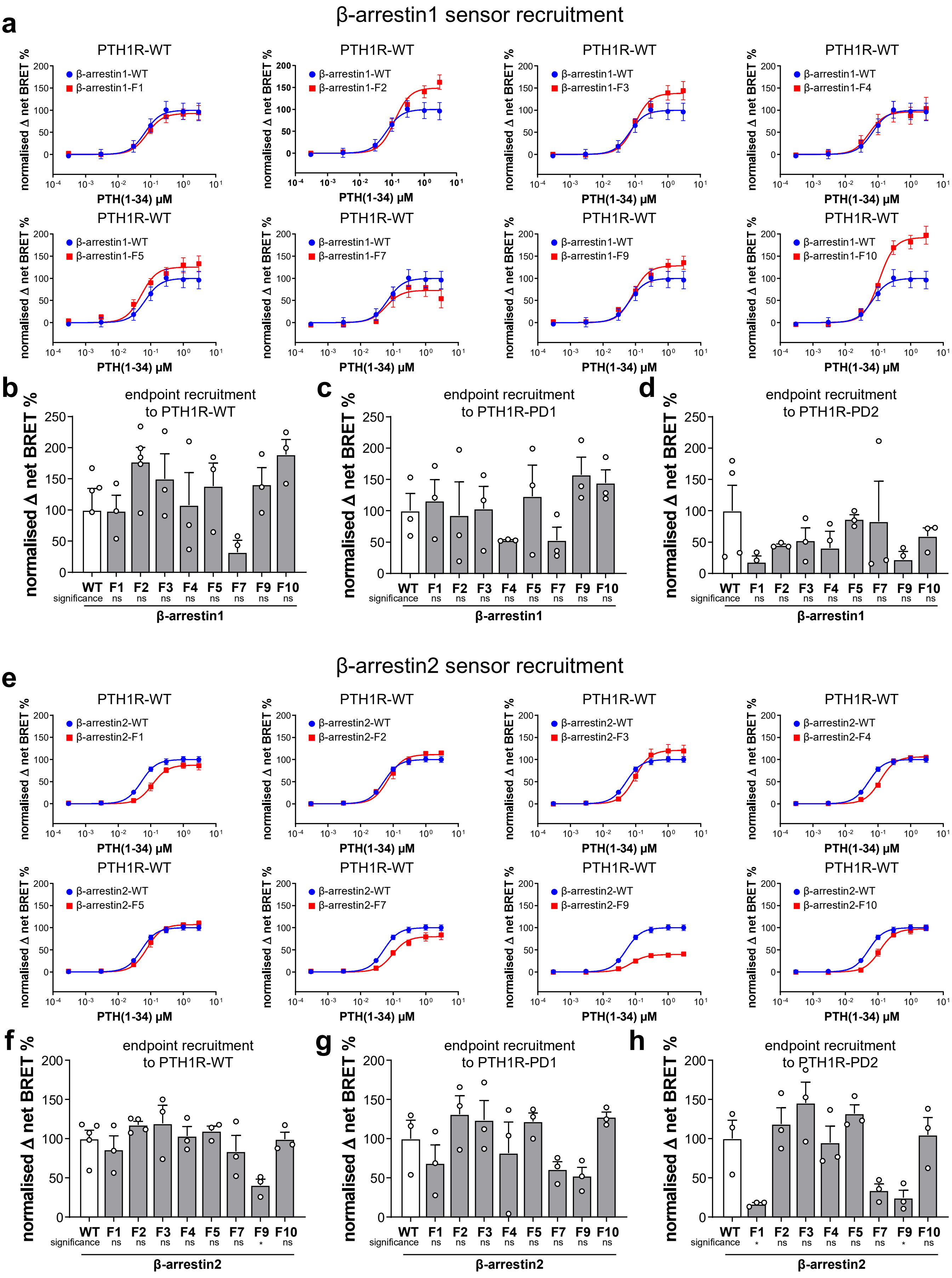

Supplementary Figure 2: Assessment of β-arrestin1 (a-d) and 2 (e-h) biosensor (F1, F2, F3, F4, F5, F7, F9 and F10) recruitment to the PTH1R-WT (a, b and e, f) and two phosphorylation-deficient receptor mutants, PTH1R-PD1 (c and g) and PTH1R-PD2 (d and h) via intermolecular NanoBRET and upon stimulation with PTH(1-34). Concentration-dependent recruitment is shown for the conditions featuring the PTH1R-WT, with data recorded for the β-arrestin-WT constructs shown multiple times to ensure comparability. Additionally, the BRET changes recorded at saturating ligand concentrations are depicted as bar charts for all three receptor variants, normalised to the respective β-arrestin-WT recruitment (b-d and f-h). Data are shown as Δ net BRET change in per cent, mean of at least three independent repetitions (β-arrestin1-WT + PTH1R-WT, and -PD2 n=4, β-arrestin1-F2 + PTH1R-WT n=5, β-arrestin2-WT + PTH1R-WT n=5, β-arrestin2-F2 + PTH1R-WT n=4, all other conditions n=3, note that some data points lie outside of axis limits) ± SEM. The statistical significance in comparison to the respective β-arrestin-WT was calculated by one-way ANOVA, followed by a one-sided Dunnett's test (*, p<0.05; **, p<0.01; ***, p<0.001; ****, p<0.0001). Exact p-values for conditions with significant differences in comparison to the respective β-arrestin-WT are: β-arrestin2-F9 + PTH1R-WT p=0.0189, β-arrestin2-F1 + PTH1R-PD2 p=0.0253, β-arrestin2-F9 + PTH1R-PD2 p=0.0471, differences for all other conditions have been found to be non significant (ns).

Source data is provided as a source data file.

Supplementary Figure 3

a

β-arrestin1 conformational changes for the interaction with PTH1R-WT, -PD1, -PD2

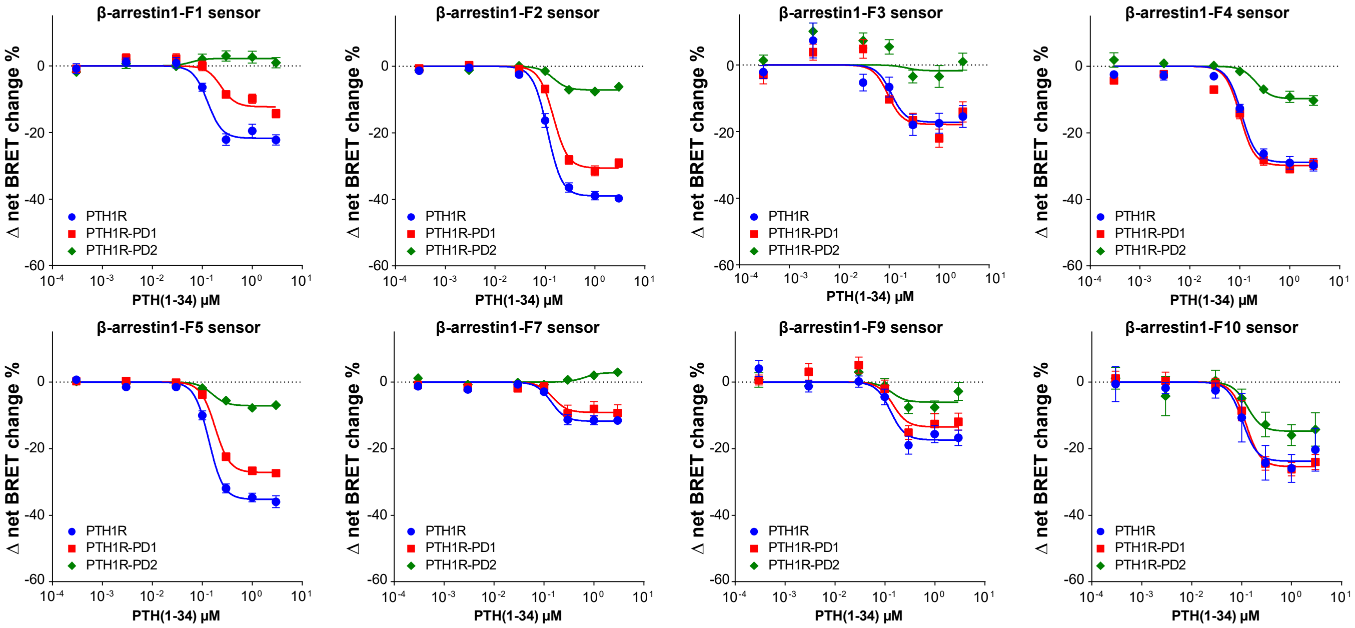

b

β-arrestin2 conformational changes for the interaction with PTH1R-WT, -PD1, -PD2

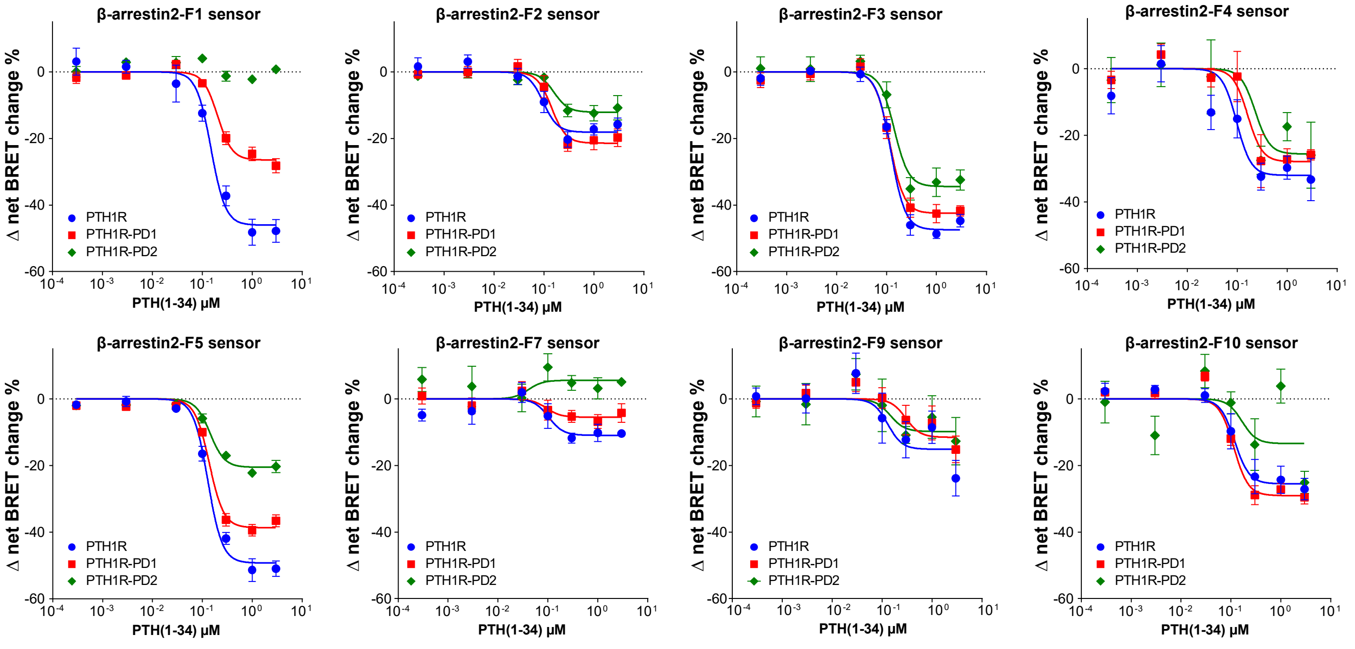

Supplementary Figure 3: Data shown corresponds to bar graphs depicted in Figure 3 and 5. Concentration dependent conformational change data for β-arrestin1 (a) and 2 (b) biosensors interacting with untagged PTH1R-WT, -PD1, PD2 variants after FlAsH-labelling and stimulation with PTH(1-34). Data are shown as Δ net BRET change in per cent, mean of three independent repetitions (n=3) ± SEM. Source data is provided as a source data file.

Supplementary Figure 4

a

β-arrestin1 conformational changes for the interaction with PTH1R-WT in ΔQ-GRK

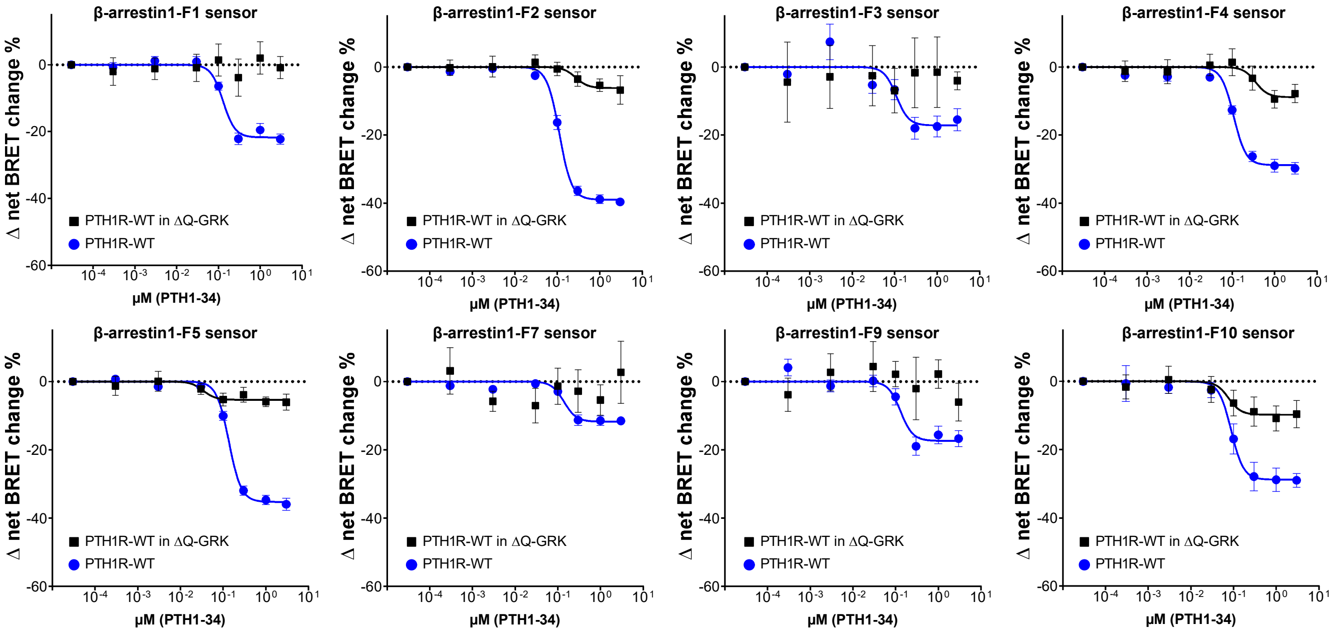

b

β-arrestin2 conformational changes for the interaction with PTH1R-WT in ΔQ-GRK

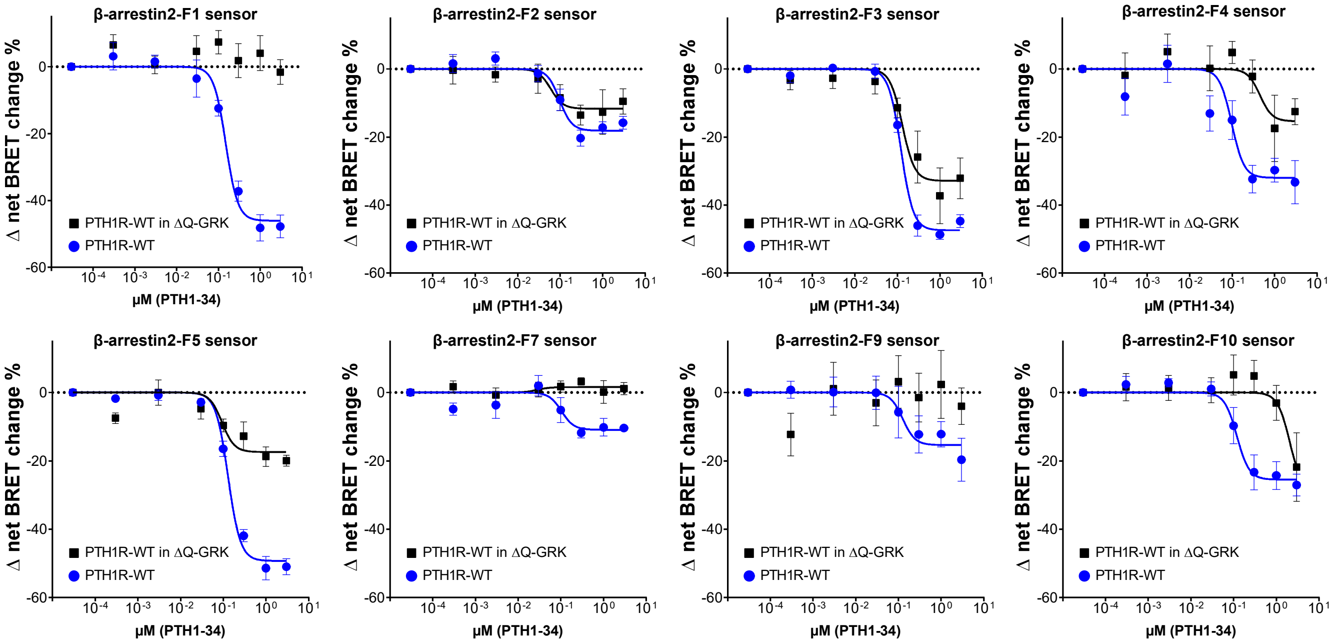

Supplementary Figure 4: Data shown corresponds to bar graphs depicted in Figure 5. Concentration-dependent conformational change data for β-arrestin1 (a) and 2 (b) biosensors interacting with untagged PTH1R-WT in the absence of GRKs using ΔQ-GRK cells after FlAsH-labelling and stimulation with PTH(1-34). PTH1R-WT curves recorded in HEK-WT (Supplementary Figure 3) are depicted again to ensure comparability. Data are shown as Δ net BRET change in per cent, mean of at least three independent repetitions (measurements in HEK-WT n=3, measurements in ΔQ-GRK n=4) ± SEM. Source data is provided as a source data file.

Supplementary Figure 5

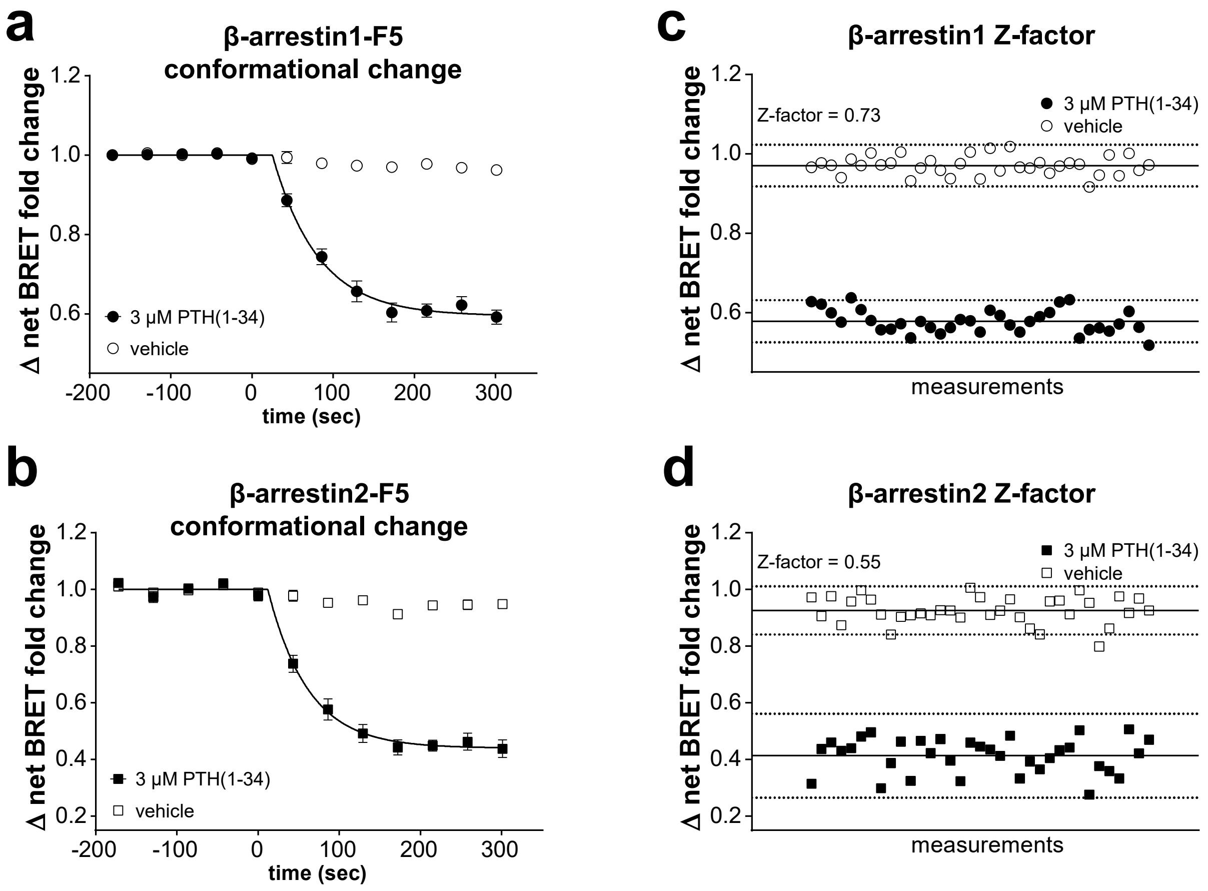

Supplementary Figure 5: a and b show the time-dependent conformational change of β-arrestin1/2-F5 upon coupling to the PTH1R-WT induced by application of 3 µM PTH(1-34) or vehicle at second zero. Data are shown as Δ net BRET change in per cent, mean of three independent repetitions (n=3, three wells per independent transfection) ± SEM. To assess the inherent Z-factor of the applied assay, data points for β-arrestin1 and 2 recorded after reaching the plateau signal are plotted individually in c and d, respectively. Additionally, lines indicating the mean and three times the standard deviation are depicted to evaluate the separation band between vehicle and 3 µM PTH(1-34)-stimulated samples. The calculated Z-factors resulting from those measurements are depicted accordingly. Source data is provided as a source data file.

Supplementary Figure 6

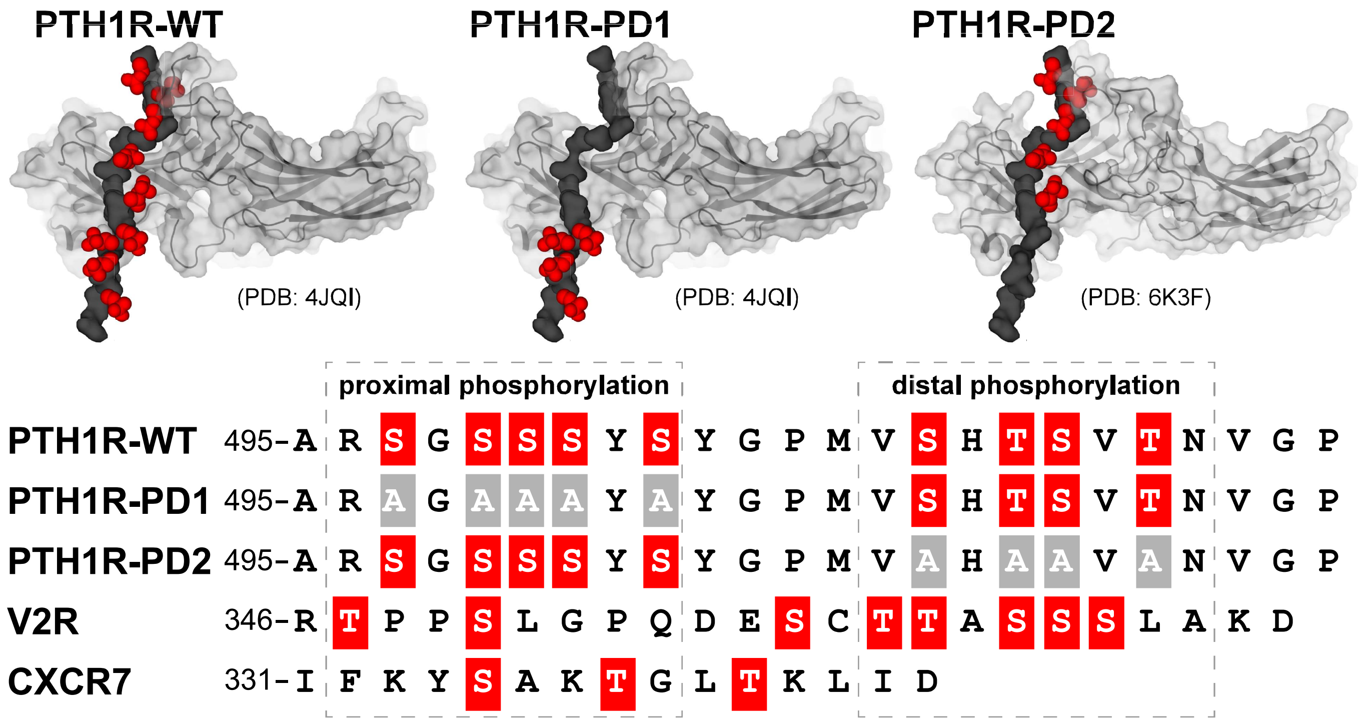

Supplementary Figure 6: Mechanistic model of β-arrestin interactions with PTH1R-WT, -PD1 and -PD2, utilising crystal structures of complexes with the V2pp (PDB: 4JQI)[1] and the CXCR7pp (PDB: 6K3F)[2] as well as a C-terminal alignment.

Supplementary Figure 7

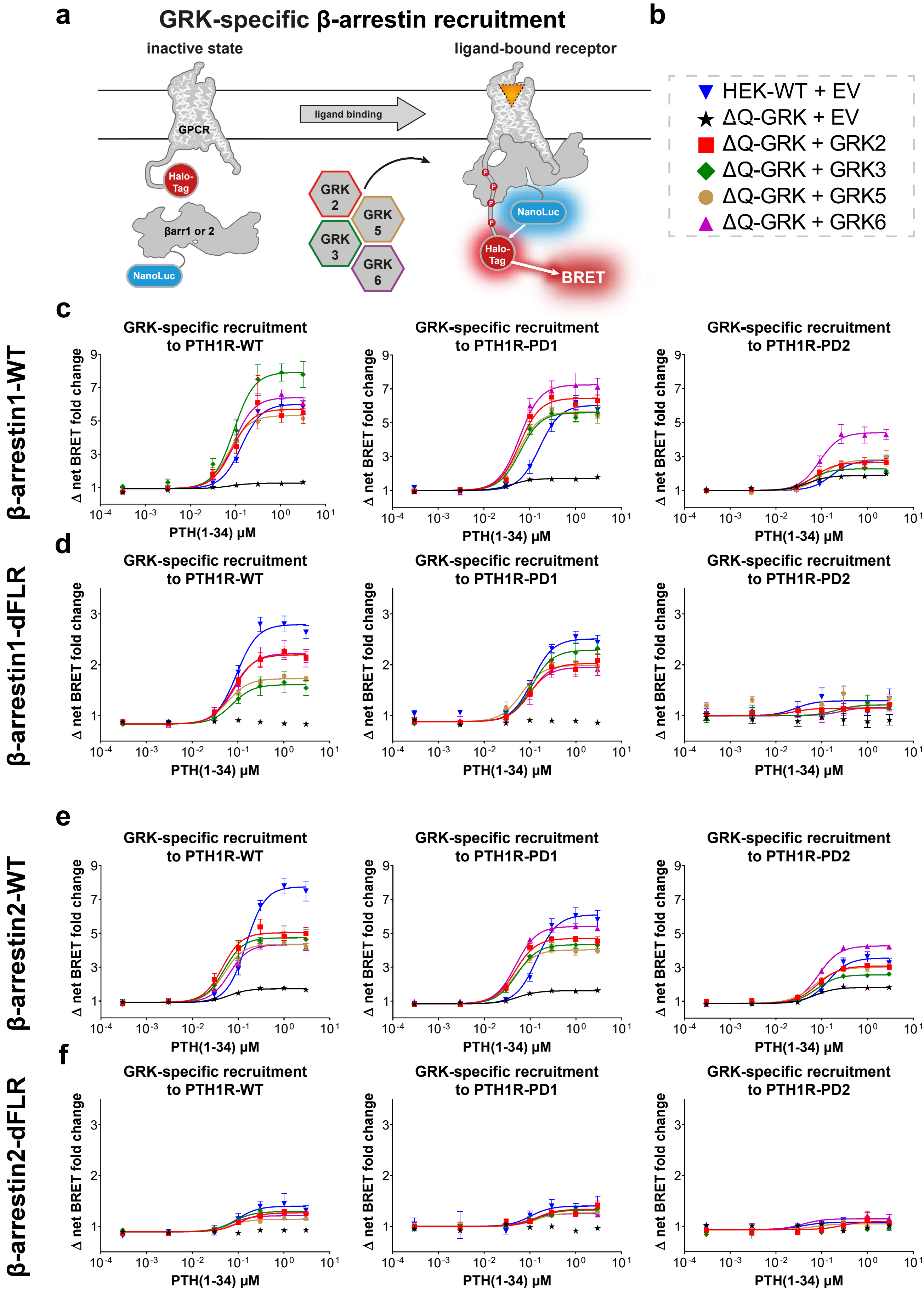

Supplementary Figure 7: a Schematic depiction of the utilised GRK-specific NanoBRET β-arrestin recruitment assay. b Colour-coding for GRK-specific conditions used in the performed assay. ΔQ-GRK or HEK-WT cells were transfected with C-terminally Halo-tagged PTH1R-WT, -PD1, -PD2 variants and β-arrestin1 (c), β-arrestin1-dFLR (d), β-arrestin2 (e), or β-arrestin2-dFLR (f) -NanoLuc fusion constructs. Additionally, either GRK2, 3, 5, 6, or the empty vector (EV) were co-transfected. The BRET changes are shown as ligand concentration-response curves. All data points are calculated as Δ net BRET fold change as the mean of three independent measurements (n=3) ± SEM. Source data is provided as a source data file.

Supplementary Figure 8

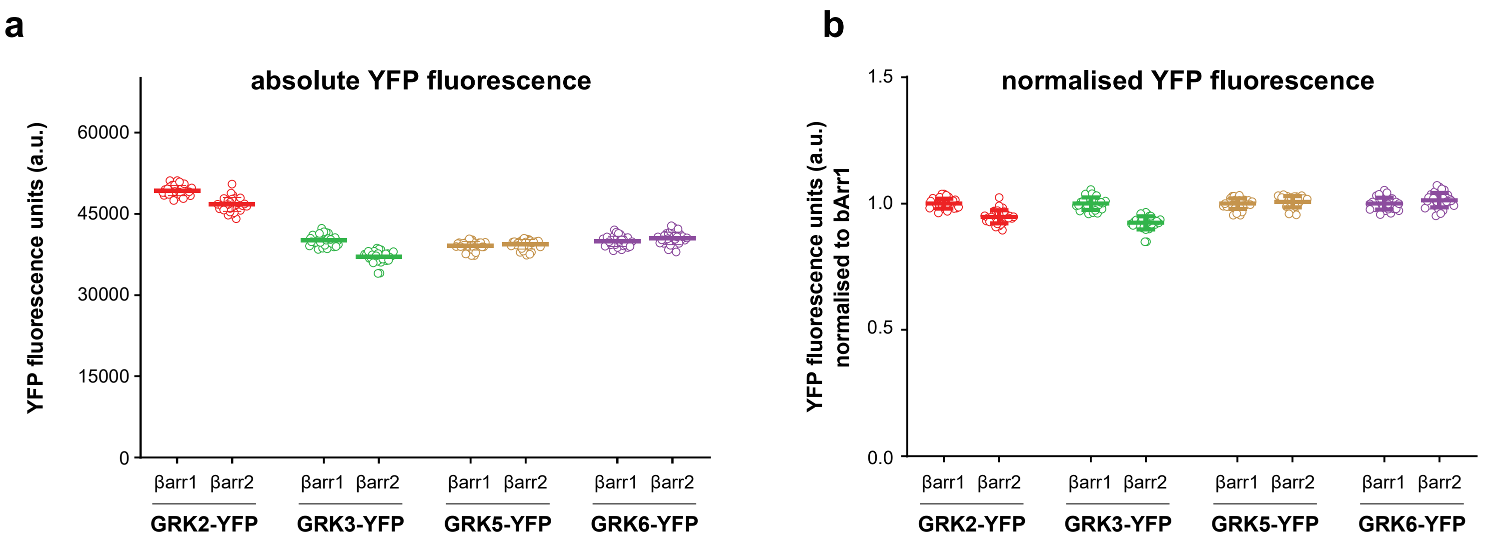

Supplementary Figure 8: GRK-YFP fusion proteins were transfected alongside the PTH1R and either β-arrestin1 and 2 in ΔQ-GRK (analogous transfection, as performed for experiments shown in Figure 4c-f and Supplementary Figure 7) and YFP fluorescence was measured to confirm similar expression levels of all transfected GRKs between the β-arrestin1 and 2 conditions. Data points represent 24 technical replicates (n=1) per transfection, while a depicts the absolute measured YFP fluorescence in arbitrary units (a.u.) and b shows values, which were normalised to the respective β-arrestin1 condition. Additional validation of protein expression levels for the GRK-specific β-arrestin recruitment assay can be found in the original publication[3] and in Reichel *et al.*[4].

Source data is provided as a source data file.

Supplementary Figure 9

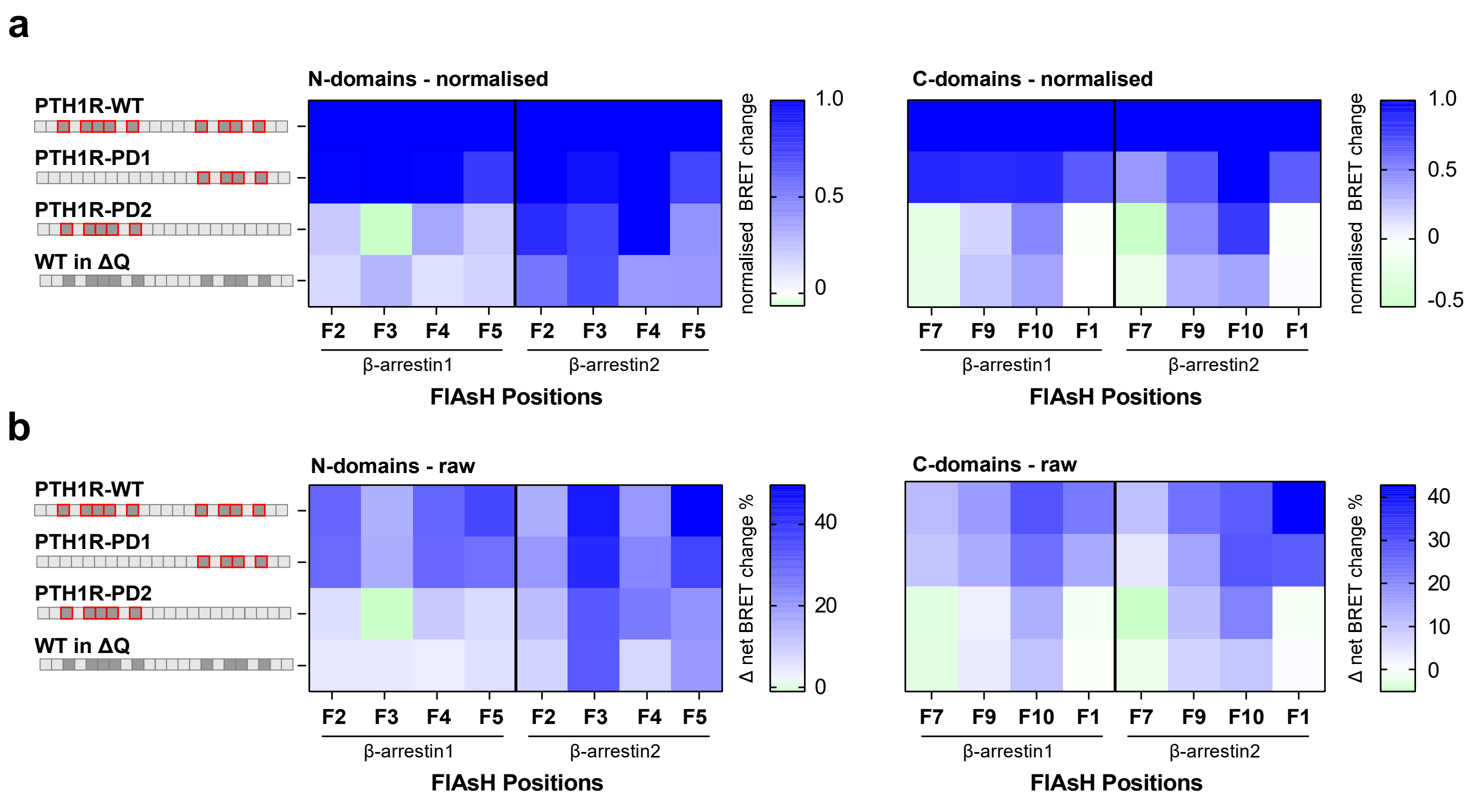

Supplementary Figure 9: Heatmap representation of the phosphorylation-specific conformational change measurements for β-arrestin1 and 2 coupling to the PTH1R-WT, PTH1R-PD1, PTH1R-PD2 and the PTH1R-WT in ΔQ-GRK, presented in Figure 5. The sensors have again been sorted into the ones that are located in the respective N- (left panels) and C-domains (right panels). a shows the BRET changes after each sensor was normalised to the respective signal induced by the PTH1R-WT. Converted Δ net BRET changes in per cent are depicted in b to enable the comparison of the initial fingerprints. Source data is provided as a source data file.

Supplementary Figure 10

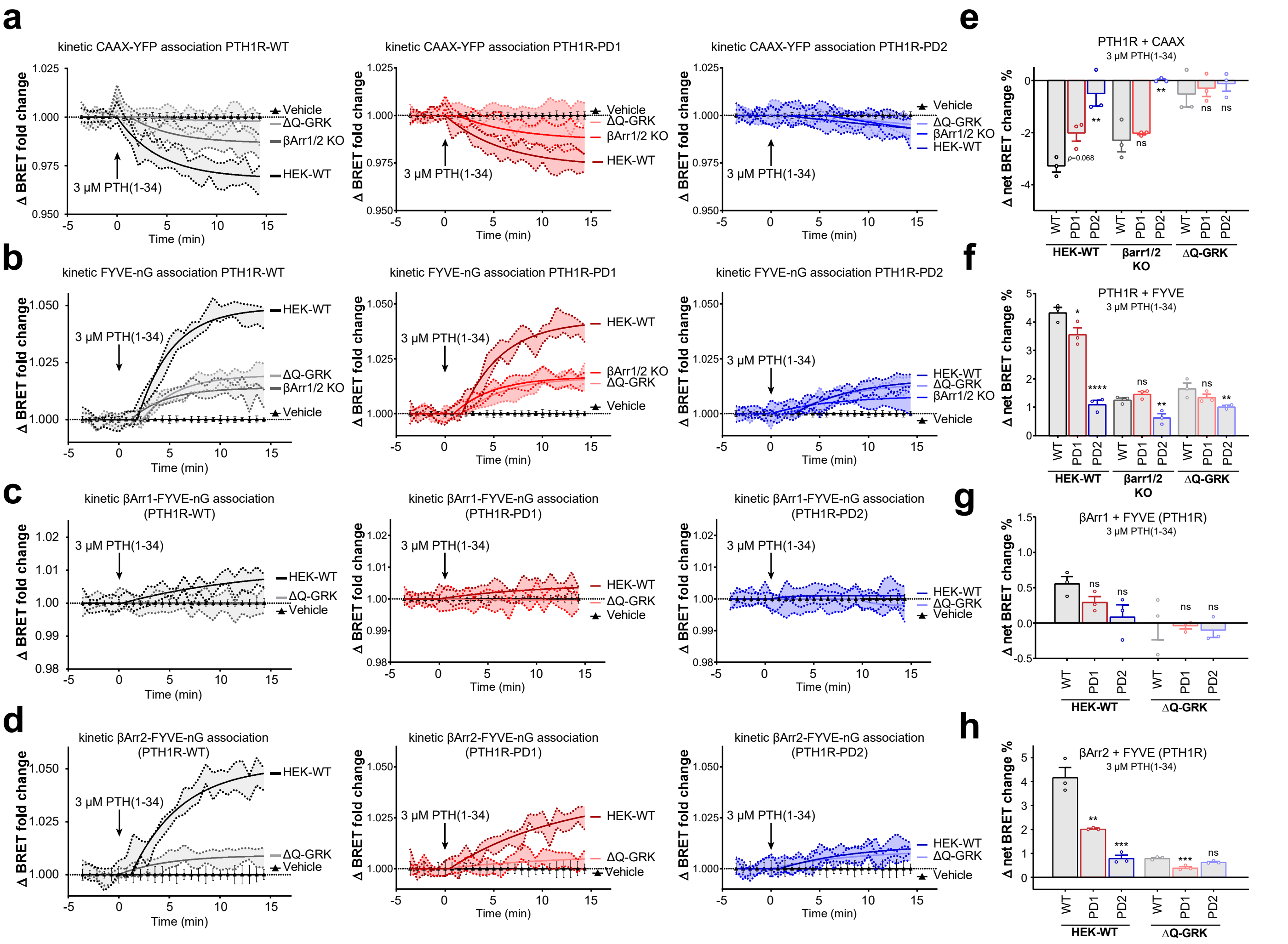

Supplementary Figure 10: a, b, c, d Time-dependent analysis of Intermolecular BRET between NanoLuc-tagged PTH1R-WT, -PD1, -PD2, or β-arrestin1 and 2 with CAAX-YFP (a) or FYVE-mNeonGreen (b, c, d), measured in HEK-WT, β-arrestin1/2 knockout cells and ΔQ-GRK. The panels correspond to Figure 6c-f and show the kinetic progression of the measurements upon receptor stimulation with 3 μM PTH(1-34). Results are shown as mean of three independent repetitions (n=3) and 95% confidence intervals (coloured areas and error bars in the case of vehicle addition). e, f, g, h Data correspond to the BRET changes at 3 μM PTH(1-34) from Figure 6c-f. Results are shown as mean of three independent repetitions (n=3) ± SEM. To test whether the specific receptor phosphorylation states significantly differ from PTH1R-WT in HEK-WT cells or the probed knockout conditions, a one-way ANOVA and two-sided Dunnett's test was performed (*, $p<0.05$; **, $p<0.01$; ***, $p<0.001$; ****, $p<0.0001$). Exact p-values for conditions with significant differences in comparison to the respective PTH1R-WT measurements were calculated as follows. b: PTH1R-PD2 in HEK-WT p=0.0021, PTH1R-PD2 in βarr1/2 KO p=0.0011; d: PTH1R-PD1 in HEK-WT p=0.0487, PTH1R-PD2 in HEK-WT p<0.0001, PTH1R-PD2 in βarr1/2 KO p=0.009, PTH1R-PD2 in ΔQ-GRK p=0.0023; h: PTH1R-PD1 in HEK-WT p=0.0014, PTH1R-PD2 in HEK-WT p=0.0001, PTH1R-PD1 in ΔQ-GRK p=0.0009. Source data is provided as a source data file.

Supplementary Figure 11

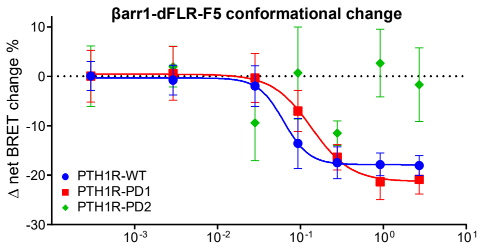

Supplementary Figure 11: Concentration-dependent conformational change of β-arrestin1-dFLR, labelled in position F5 upon activation of either the PTH1R-WT, -PD1 or PD2 variants with PTH(1-34). Results are shown as mean of three independent repetitions (n=3) ± SEM. Source data is provided as a source data file.

Supplementary Table 1: Results of the statistical analysis of co-localisation between PTH1R-CFP and Rab5-mCherry, as presented in Figure 1f and i. For each indicated condition the co-localisation before stimulation (baseline) and 15 minutes after ligand application (stim) was compared using two-way ANOVA, followed by a post-hoc comparison with Bonferroni correction (* $p < 0.05$; ** $p < 0.01$; *** $p < 0.001$; **** $p < 0.0001$). Additionally shown are the mean difference and 95% confidence intervals (CI) of the difference.

conditions	mean diff.	95.00% CI of diff.	*p* value	
bArr1_baseline vs. bArr1_stim	-1.311	-1.803 to -0.8192	<0.0001	****
bArr1_baseline vs. bArr1-dFLR_baseline	0.000	-0.5067 to 0.5067	>0.9999	ns
bArr1_baseline vs. bArr1-dFLR_stim	-0.952	-1.441 to -0.4624	<0.0001	****
bArr1_baseline vs. no bArr_baseline	0.000	-0.6659 to 0.6659	>0.9999	ns
bArr1_baseline vs. no bArr_stim	-0.541	-1.182 to 0.09961	0.2289	ns
bArr1_baseline vs. bArr1+dQ_baseline	0.000	-0.5223 to 0.5223	>0.9999	ns
bArr1_baseline vs. bArr1+dQ_stim	-0.243	-0.7614 to 0.2763	>0.9999	ns
bArr1_stim vs. bArr1-dFLR_baseline	1.311	0.8321 to 1.79	<0.0001	****
bArr1_stim vs. bArr1-dFLR_stim	0.359	-0.1016 to 0.8196	0.4081	ns
bArr1_stim vs. no bArr_baseline	1.311	0.6659 to 1.956	<0.0001	****
bArr1_stim vs. no bArr_stim	0.769	0.1502 to 1.389	0.0031	**
bArr1_stim vs. bArr1+dQ_baseline	1.311	0.8156 to 1.806	<0.0001	****
bArr1_stim vs. bArr1+dQ_stim	1.068	0.5766 to 1.56	<0.0001	****
bArr1-dFLR_baseline vs. bArr1-dFLR_stim	-0.952	-1.428 to -0.4753	<0.0001	****
bArr1-dFLR_baseline vs. no bArr_baseline	0.000	-0.6564 to 0.6564	>0.9999	ns
bArr1-dFLR_baseline vs. no bArr_stim	-0.541	-1.173 to 0.08977	0.2028	ns
bArr1-dFLR_baseline vs. bArr1+dQ_baseline	0.000	-0.5101 to 0.5101	>0.9999	ns
bArr1-dFLR_baseline vs. bArr1+dQ_stim	-0.243	-0.7492 to 0.2641	>0.9999	ns
bArr1-dFLR_stim vs. no bArr_baseline	0.952	0.3086 to 1.595	0.0001	***
bArr1-dFLR_stim vs. no bArr_stim	0.411	-0.207 to 1.028	>0.9999	ns
bArr1-dFLR_stim vs. bArr1+dQ_baseline	0.952	0.4588 to 1.445	<0.0001	****
bArr1-dFLR_stim vs. bArr1+dQ_stim	0.709	0.2198 to 1.199	0.0002	***
no bArr_baseline vs. no bArr_stim	-0.541	-1.306 to 0.2235	0.739	ns
no bArr_baseline vs. bArr1+dQ_baseline	0.000	-0.6686 to 0.6686	>0.9999	ns
no bArr_baseline vs. bArr1+dQ_stim	-0.243	-0.9084 to 0.4234	>0.9999	ns
no bArr_stim vs. bArr1+dQ_baseline	0.541	-0.1024 to 1.185	0.2366	ns
no bArr_stim vs. bArr1+dQ_stim	0.299	-0.3422 to 0.9399	>0.9999	ns
bArr1+dQ_baseline vs. bArr1+dQ_stim	-0.243	-0.7648 to 0.2797	>0.9999	ns
bArr2_baseline vs. bArr2_stim	-1.211	-1.709 to -0.7121	<0.0001	****
bArr2_baseline vs. bArr2-dFLR_baseline	-3.03E-10	-0.5334 to 0.5334	>0.9999	ns
bArr2_baseline vs. bArr2-dFLR_stim	-0.5697	-1.068 to -0.07097	0.0104	*
bArr2_baseline vs. no bArr_baseline	-3.03E-10	-0.6136 to 0.6136	>0.9999	ns
bArr2_baseline vs. bArr1_stim	-0.5414	-1.133 to 0.05048	0.118	ns
bArr2_baseline vs. bArr2+dQ_baseline	-3.03E-10	-0.461 to 0.461	>0.9999	ns
bArr2_baseline vs. bArr2+dQ_stim	-0.1502	-0.6028 to 0.3023	>0.9999	ns
bArr2_stim vs. bArr2-dFLR_baseline	1.211	0.6843 to 1.737	<0.0001	****
bArr2_stim vs. bArr2-dFLR_stim	0.6411	0.1498 to 1.132	0.0014	**
bArr2_stim vs. no bArr_baseline	1.211	0.6032 to 1.818	<0.0001	****
bArr2_stim vs. bArr1_stim	0.6694	0.08369 to 1.255	0.0104	*
bArr2_stim vs. bArr2+dQ_baseline	1.211	0.7578 to 1.664	<0.0001	****
bArr2_stim vs. bArr2+dQ_stim	1.061	0.6161 to 1.505	<0.0001	****
bArr2-dFLR_baseline vs. bArr2-dFLR_stim	-0.5697	-1.096 to -0.04322	0.0207	*
bArr2-dFLR_baseline vs. no bArr_baseline	0	-0.6363 to 0.6363	>0.9999	ns
bArr2-dFLR_baseline vs. bArr1_stim	-0.5414	-1.157 to 0.07404	0.165	ns
bArr2-dFLR_baseline vs. bArr2+dQ_baseline	0	-0.4909 to 0.4909	>0.9999	ns
bArr2-dFLR_baseline vs. bArr2+dQ_stim	-0.1502	-0.6332 to 0.3327	>0.9999	ns
bArr2-dFLR_stim vs. no bArr_baseline	0.5697	-0.03793 to 1.177	0.0943	ns
bArr2-dFLR_stim vs. bArr1_stim	0.02825	-0.5574 to 0.6139	>0.9999	ns
bArr2-dFLR_stim vs. bArr2+dQ_baseline	0.5697	0.1167 to 1.023	0.0026	**
bArr2-dFLR_stim vs. bArr2+dQ_stim	0.4194	-0.02498 to 0.8638	0.0887	ns
no bArr_baseline vs. bArr1_stim	-0.5414	-1.228 to 0.1447	0.3747	ns
no bArr_baseline vs. bArr2+dQ_baseline	0	-0.577 to 0.577	>0.9999	ns
no bArr_baseline vs. bArr2+dQ_stim	-0.1502	-0.7206 to 0.4201	>0.9999	ns
bArr1_stim vs. bArr2+dQ_baseline	0.5414	-0.01249 to 1.095	0.0631	ns
bArr1_stim vs. bArr2+dQ_stim	0.3912	-0.1558 to 0.9381	0.6948	ns
bArr2+dQ_baseline vs. bArr2+dQ_stim	-0.1502	-0.5519 to 0.2514	>0.9999	ns

Supplementary Table 2: EC_{50} values and standard errors derived from conformational change measurements of individual β-arrestin1 FlAsH biosensors upon stimulation of the respective PTH1R phosphorylation-specific receptor state with PTH(1-34) in µM.

β-arrestin1	PTH1R-WT	PTH1R-PD1	PTH1R-PD2	PTH1R in ΔQ-GRK
**F1**	0.126 ± 0.015	0.217 ± 0.039	N/A	N/A
**F2**	0.115 ± 0.006	0.149 ± 0.010	0.159 ± 0.048	0.270 ± 0.160
**F3**	0.107 ± 0.030	0.088 ± 0.018	0.146 ± 0.080	N/A
**F4**	0.120 ± 0.010	0.117 ± 0.010	0.184 ± 0.052	0.356 ± 0.187
**F5**	0.140 ± 0.009	0.179 ± 0.011	0.188 ± 0.037	0.034 ± 0.019
**F7**	0.157 ± 0.032	0.168 ± 0.074	0.473 ± 0.324	N/A
**F9**	0.123 ± 0.025	0.124 ± 0.030	0.125 ± 0.058	N/A
**F10**	0.110 ± 0.037	0.122 ± 0.017	0.141 ± 0.073	0.076 ± 0.046

Supplementary Table 3: EC_{50} values and standard errors derived from conformational change measurements of individual β-arrestin2 FlAsH biosensors upon stimulation of the respective PTH1R phosphorylation-specific receptor state with PTH(1-34) in µM.

β-arrestin2	PTH1R-WT	PTH1R-PD1	PTH1R-PD2	PTH1R in ΔQ-GRK
**F1**	0.160 ± 0.025	0.202 ± 0.024	N/A	N/A
**F2**	0.088 ± 0.019	0.140 ± 0.028	0.172 ± 0.061	0.096 ± 0.017
**F3**	0.125 ± 0.010	0.119 ± 0.012	0.136 ± 0.025	0.122 ± 0.008
**F4**	0.119 ± 0.042	0.162 ± 0.057	0.230 ± 0.330	0.098 ± 0.022
**F5**	0.144 ± 0.011	0.149 ± 0.011	0.175 ± 0.023	0.093 ± 0.022
**F7**	0.126 ± 0.071	0.092 ± 0.066	N/A	N/A
**F9**	0.112 ± 0.057	0.266 ± 0.127	N/A	N/A
**F10**	0.112 ± 0.026	0.104 ± 0.011	N/A	N/A

Supplementary Table 4: Results of the statistical analysis of conformational change measurement presented. For each indicated condition the BRET ratio after ligand application in the highest concentration was compared to the recorded BRET ratio after vehicle stimulation using two-sided Student's t-test (* $p < 0.05$; ** $p < 0.01$; *** $p < 0.001$). Additionally shown are the effect size, lower and upper confidence intervals (CI), t-statistic and degrees of freedom (df).

condition	sensor	effect size	lower 95% CI	upper 95% CI	t statistic	df	*p* value	
PTH1R-βArr1	F1	-6.084	-0.302	-0.138	-7.451	3.997	0.0017	**
PTH1R-βArr1	F2	-2.374	-0.704	0.135	-2.908	2.004	0.1005	
PTH1R-βArr1	F3	-8.036	-0.197	-0.109	-9.842	3.875	0.0007	***
PTH1R-βArr1	F4	-9.483	-0.431	-0.202	-11.614	2.048	0.0067	**
PTH1R-βArr1	F5	-8.521	-0.487	-0.208	-10.435	2.060	0.0082	**
PTH1R-βArr1	F7	-2.837	-0.220	-0.019	-3.474	3.558	0.0307	*
PTH1R-βArr1	F9	-3.796	-0.255	-0.084	-5.368	4.247	0.0049	**
PTH1R-βArr1	F10	-2.243	-0.470	0.051	-2.747	2.675	0.0806	
PTH1R-βArr2	F1	-3.931	-0.718	-0.075	-4.854	2.211	0.0326	*
PTH1R-βArr2	F2	-2.575	-0.339	0.040	-3.174	2.154	0.0786	
PTH1R-βArr2	F3	-13.647	-0.489	-0.379	-19.299	5.984	0.0000	***
PTH1R-βArr2	F4	-1.846	-0.270	0.041	-2.261	3.187	0.1036	
PTH1R-βArr2	F5	-8.665	-0.658	-0.284	-10.632	2.045	0.0081	**
PTH1R-βArr2	F7	-1.994	-0.242	0.018	-2.442	3.834	0.0738	
PTH1R-βArr2	F9	-1.913	-0.467	0.097	-2.342	2.502	0.1183	
PTH1R-βArr2	F10	-2.457	-0.430	-0.072	-3.475	5.702	0.0144	*
ΔQ-βArr1	F1	0.840	-0.078	0.194	1.188	4.002	0.3007	
ΔQ-βArr1	F2	-0.926	-0.131	0.040	-1.310	6.000	0.2382	
ΔQ-βArr1	F3	0.183	-0.319	0.394	0.258	5.995	0.8049	
ΔQ-βArr1	F4	-0.491	-0.188	0.112	-0.694	4.124	0.5246	
ΔQ-βArr1	F5	-1.317	-0.140	0.020	-1.862	5.731	0.1142	
ΔQ-βArr1	F7	-0.317	-0.317	0.219	-0.449	5.888	0.6697	
ΔQ-βArr1	F9	0.637	-0.072	0.142	0.901	4.160	0.4166	
ΔQ-βArr1	F10	-0.560	-0.341	0.176	-0.792	5.723	0.4599	
ΔQ-βArr2	F1	-0.021	-0.192	0.188	-0.030	5.521	0.9770	
ΔQ-βArr2	F2	-1.026	-0.378	0.117	-1.451	4.100	0.2186	
ΔQ-βArr2	F3	-2.159	-0.629	0.010	-3.053	3.054	0.0540	
ΔQ-βArr2	F4	-0.064	-0.379	0.358	-0.090	3.182	0.9335	
ΔQ-βArr2	F5	-3.739	-0.296	-0.108	-5.288	5.909	0.0019	**
ΔQ-βArr2	F7	0.305	-0.186	0.260	0.432	4.732	0.6848	
ΔQ-βArr2	F9	-0.707	-0.631	0.265	-1.000	5.964	0.3561	
ΔQ-βArr2	F10	-0.937	-0.460	0.152	-1.325	4.631	0.2469	
PD1-βArr1	F1	-3.379	-0.249	-0.040	-4.139	3.350	0.0206	*
PD1-βArr1	F2	-11.221	-0.351	-0.223	-13.743	3.224	0.0006	***
PD1-βArr1	F3	-1.626	-0.429	0.140	-1.991	2.224	0.1717	
PD1-βArr1	F4	-6.730	-0.456	-0.176	-8.242	2.422	0.0079	**
PD1-βArr1	F5	-12.182	-0.337	-0.196	-14.920	2.211	0.0029	**
PD1-βArr1	F7	-1.769	-0.254	0.061	-2.166	2.539	0.1351	
PD1-βArr1	F9	-2.395	-0.215	-0.034	-3.387	5.920	0.0150	*
PD1-βArr1	F10	-4.763	-0.356	-0.126	-5.834	3.994	0.0043	**
PD1-βArr2	F1	-2.894	-0.431	-0.055	-3.721	3.683	0.0237	*
PD1-βArr2	F2	-2.309	-0.487	0.062	-2.868	2.379	0.0844	
PD1-βArr2	F3	-12.776	-0.472	-0.355	-18.068	5.075	0.0000	***
PD1-βArr2	F4	-3.758	-0.456	-0.044	-4.602	2.317	0.0331	*
PD1-βArr2	F5	-6.617	-0.514	-0.179	-8.169	2.204	0.0109	*
PD1-βArr2	F7	-2.073	-0.097	0.004	-2.539	3.974	0.0644	
PD1-βArr2	F9	-1.653	-0.402	0.113	-2.024	2.458	0.1559	
PD1-βArr2	F10	-3.782	-0.403	-0.145	-5.348	5.378	0.0025	**
PD2-βArr1	F1	0.462	-0.044	0.066	0.566	3.998	0.6016	
PD2-βArr1	F2	-2.526	-0.123	-0.002	-3.094	3.372	0.0458	*
PD2-βArr1	F3	0.388	-0.046	0.065	0.475	3.957	0.6599	
PD2-βArr1	F4	-4.179	-0.166	-0.047	-5.119	3.729	0.0083	**
PD2-βArr1	F5	-4.672	-0.101	-0.034	-5.722	3.860	0.0051	**
PD2-βArr1	F7	0.915	-0.044	0.103	1.121	4.000	0.3250	
PD2-βArr1	F9	-0.509	-0.136	0.079	-0.719	4.158	0.5104	
PD2-βArr1	F10	-0.991	-0.578	0.315	-1.214	2.096	0.3437	
PD2-βArr2	F1	0.067	-0.105	0.112	0.087	3.791	0.9353	
PD2-βArr2	F2	-1.476	-0.321	0.098	-1.847	2.603	0.1758	
PD2-βArr2	F3	-3.869	-0.482	-0.123	-4.738	3.881	0.0097	**
PD2-βArr2	F4	-2.373	-0.602	-0.009	-2.906	3.858	0.0458	*
PD2-βArr2	F5	-7.528	-0.258	-0.151	-9.959	4.801	0.0002	***
PD2-βArr2	F7	2.330	0.001	0.102	2.853	3.882	0.0479	*
PD2-βArr2	F9	-1.055	-0.303	0.111	-1.292	3.948	0.2666	
PD2-βArr2	F10	-1.170	-0.424	0.088	-1.655	5.351	0.1551	

Supplementary Table 5: Results of the statistical analysis of conformational change measurement presented in Figure 5. For each indicated phosphorylation-specific condition the BRET ratio after ligand application in the highest concentration was compared to measurements using the PTH1R-WT receptor. The statistical significance was calculated by one-way ANOVA, followed by a two-sided Dunnett's test (*, p<0.05; **, p<0.001; ***, p<0.0001; ****, p<0.00001). Additionally shown are the mean difference and 95% confidence intervals (CI) of the difference.

biosensor	multiple comparison	Mean difference	95% CI of difference	Adjusted *p-v*alue	
**βArr1-F1**	WT vs. PD1	-8,041	-18,22 to 2,134	0,1251	ns
	WT vs. PD2	-23,64	-33,82 to -13,47	0,0003	***
	WT vs. WT in dQ	-22,7	-32,21 to -13,18	0,0003	***
**βArr1-F2**	WT vs. PD1	-0,6617	-19,27 to 17,94	0,9992	ns
	WT vs. PD2	-23,34	-41,94 to -4,731	0,0165	*
	WT vs. WT in dQ	-25,13	-42,53 to -7,723	0,0074	**
**βArr1-F3**	WT vs. PD1	0,5558	-10,47 to 11,58	0,9977	ns
	WT vs. PD2	-16,53	-27,56 to -5,507	0,0059	**
	WT vs. WT in dQ	-11,09	-21,41 to -0,7798	0,0359	*
**βArr1-F4**	WT vs. PD1	-0,4785	-14,37 to 13,41	0,9993	ns
	WT vs. PD2	-19,51	-33,40 to -5,620	0,0087	**
	WT vs. WT in dQ	-26,19	-39,18 to -13,20	0,0008	***
**βArr1-F5**	WT vs. PD1	-8,242	-17,50 to 1,012	0,0809	ns
	WT vs. PD2	-28,69	-37,94 to -19,43	<0,0001	****
	WT vs. WT in dQ	-29,63	-38,29 to -20,97	<0,0001	****
**βArr1-F7**	WT vs. PD1	-1,757	-16,66 to 13,15	0,974	ns
	WT vs. PD2	-14,47	-29,37 to 0,4370	0,0569	ns
	WT vs. WT in dQ	-14,25	-28,19 to -0,3113	0,0453	*
**βArr1-F9**	WT vs. PD1	-2,798	-16,98 to 11,38	0,9114	ns
	WT vs. PD2	-13,93	-28,10 to 0,2530	0,0544	ns
	WT vs. WT in dQ	-13,04	-27,22 to 1,136	0,073	ns
**βArr1-F10**	WT vs. PD1	-4,542	-22,41 to 13,32	0,8148	ns
	WT vs. PD2	-15,2	-33,07 to 2,664	0,0963	ns
	WT vs. WT in dQ	-18,48	-35,19 to -1,764	0,0314	*
**βArr2-F1**	WT vs. PD1	-15,94	-33,30 to 1,408	0,0715	ns
	WT vs. PD2	-43,86	-61,21 to -26,51	0,0002	***
	WT vs. WT in dQ	-42,49	-58,72 to -26,26	0,0001	***
**βArr2-F2**	WT vs. PD1	4,418	-12,59 to 21,43	0,8058	ns
	WT vs. PD2	-2,666	-19,67 to 14,34	0,9434	ns
	WT vs. WT in dQ	-7,236	-23,15 to 8,673	0,4694	ns
**βArr2-F3**	WT vs. PD1	-2,961	-18,95 to 13,03	0,9237	ns
	WT vs. PD2	-12,31	-29,58 to 4,962	0,1835	ns
	WT vs. WT in dQ	-12,82	-28,81 to 3,171	0,1243	ns
**βArr2-F4**	WT vs. PD1	3,522	-14,83 to 21,88	0,9047	ns
	WT vs. PD2	5,813	-12,54 to 24,17	0,708	ns
	WT vs. WT in dQ	-12,02	-29,19 to 5,152	0,1835	ns
**βArr2-F5**	WT vs. PD1	-12,94	-24,32 to -1,565	0,0273	*
	WT vs. PD2	-28,87	-40,24 to -17,49	0,0002	***
	WT vs. WT in dQ	-29,65	-40,30 to -19,01	<0,0001	****
**βArr2-F7**	WT vs. PD1	-6,231	-15,18 to 2,716	0,1862	ns
	WT vs. PD2	-15,55	-24,49 to -6,600	0,0023	**
	WT vs. WT in dQ	-12,44	-20,81 to -4,070	0,0062	**
**βArr2-F9**	WT vs. PD1	-8,531	-27,53 to 10,47	0,4786	ns
	WT vs. PD2	-12,98	-31,98 to 6,021	0,1968	ns
	WT vs. WT in dQ	-16,48	-34,25 to 1,297	0,0689	ns
**βArr2-F10**	WT vs. PD1	1,087	-12,78 to 14,95	0,9932	ns
	WT vs. PD2	-6,252	-20,12 to 7,613	0,5048	ns
	WT vs. WT in dQ	-17,6	-31,47 to -3,740	0,0136	*

Supplementary References

1 Shukla, A. K. et al. Structure of active beta-arrestin-1 bound to a G-protein-coupled receptor phosphopeptide. Nature 497, 137-141, doi:10.1038/nature12120 (2013).

2 Min, K. et al. Crystal Structure of beta-Arrestin 2 in Complex with CXCR7 Phosphopeptide. Structure, doi:10.1016/j.str.2020.06.002 (2020).

3 Drube, J. et al. GPCR kinase knockout cells reveal the impact of individual GRKs on arrestin binding and GPCR regulation. Nat Commun 13, 540, doi:10.1038/s41467-022-28152-8 (2022).

4 Reichel, M., Weitzel, V., Klement, L., Hoffmann, C. & Drube, J. Suitability of GRK Antibodies for Individual Detection and Quantification of GRK Isoforms in Western Blots. Int J Mol Sci 23, doi:10.3390/ijms23031195 (2022).
