# Supplementary material for: β-arrestin1 and 2 exhibit distinct phosphorylation-dependent conformations when coupling to the same GPCR in living cells
Source: Nat Commun. 2022 Sep 26;13:5638. doi: 10.1038/s41467-022-33307-8 (PMC9512828; doi:10.1038/s41467-022-33307-8)
Supplement: Supplementary file 9 — Reporting Summary [file 41467_2022_33307_MOESM9_ESM.pdf]

nature portfolio

Corresponding author(s): Hoffmann, Carsten

Last updated by author(s): Aug 15, 2022

# Reporting Summary

Nature Portfolio wishes to improve the reproducibility of the work that we publish. This form provides structure for consistency and transparency in reporting. For further information on Nature Portfolio policies, see our Editorial Policies and the Editorial Policy Checklist.

## Statistics

For all statistical analyses, confirm that the following items are present in the figure legend, table legend, main text, or Methods section.

| n/a | Confirmed | |
|---|---|---|
| ☐ | ☒ | The exact sample size (*n*) for each experimental group/condition, given as a discrete number and unit of measurement |
| ☐ | ☒ | A statement on whether measurements were taken from distinct samples or whether the same sample was measured repeatedly |
| ☐ | ☒ | The statistical test(s) used AND whether they are one- or two-sided<br>*Only common tests should be described solely by name; describe more complex techniques in the Methods section.* |
| ☒ | ☐ | A description of all covariates tested |
| ☐ | ☒ | A description of any assumptions or corrections, such as tests of normality and adjustment for multiple comparisons |
| ☐ | ☒ | A full description of the statistical parameters including central tendency (e.g. means) or other basic estimates (e.g. regression coefficient) AND variation (e.g. standard deviation) or associated estimates of uncertainty (e.g. confidence intervals) |
| ☐ | ☒ | For null hypothesis testing, the test statistic (e.g. *F*, *t*, *r*) with confidence intervals, effect sizes, degrees of freedom and *P* value noted<br>*Give P values as exact values whenever suitable.* |
| ☒ | ☐ | For Bayesian analysis, information on the choice of priors and Markov chain Monte Carlo settings |
| ☒ | ☐ | For hierarchical and complex designs, identification of the appropriate level for tests and full reporting of outcomes |
| ☒ | ☐ | Estimates of effect sizes (e.g. Cohen's *d*, Pearson's *r*), indicating how they were calculated |

*Our web collection on statistics for biologists contains articles on many of the points above.*

## Software and code

Policy information about availability of computer code

| | |
|---|---|
| Data collection | Live cell microscopy data was obtained using an Leica SP8 with the Leica Application Suite X, Version 3.5.5.19976; plate reader data was obtained by using a BioTek Synergy Neo2 platereader with the Gen5 software Version 2.09; Quantification of western blot images was done using Fujifilm multi Gauge software (V3.0). |
| Data analysis | For data analysis Microsoft office Excel version 2010, Graphpad Prism 7, ImageJ Version 1.52p, the Squassh (Version 1) and SquasshAnalyst (Version 1) Software as described in the Methods section and references by A. Rizk et al.; PyMOL Version 2.5.2 was used to display protein structures. |

For manuscripts utilizing custom algorithms or software that are central to the research but not yet described in published literature, software must be made available to editors and reviewers. We strongly encourage code deposition in a community repository (e.g. GitHub). See the Nature Portfolio guidelines for submitting code & software for further information.

## Data

Policy information about availability of data

All manuscripts must include a data availability statement. This statement should provide the following information, where applicable:

- Accession codes, unique identifiers, or web links for publicly available datasets
- A description of any restrictions on data availability
- For clinical datasets or third party data, please ensure that the statement adheres to our policy

The following protein structures were used in this manuscirpt:
o β-arrestin1 in complex with V2pp PDB 4JQI
o β-arrestin2 in complex with CXCR7pp PDB 6K3F

o PTH1R in complex with Gs PDB 6NBF
o arrestin-1 PDB 1CF1
o active arrestin-1 PDB 5W0P
o inactive β-arrestin1 PDB 2WTR
o inactive β-arrestin2 PDB 3P2D

Source data are provided with this paper.

# Field-specific reporting

Please select the one below that is the best fit for your research. If you are not sure, read the appropriate sections before making your selection.

- [x] Life sciences
- [ ] Behavioural & social sciences
- [ ] Ecological, evolutionary & environmental sciences

For a reference copy of the document with all sections, see nature.com/documents/nr-reporting-summary-flat.pdf

# Life sciences study design

All studies must disclose on these points even when the disclosure is negative.

| | |
|---|---|
| Sample size | No sample size calculation was performed. Our platereader experiments were carried out in three (or more where indicated) independent experiments (independent transfections and readings) with each well representing more than 10Exp4 cells and each datapoint measured in triplicates, based on other studies with similiar methodology (PMID: 27397672; PMID: 35078997). The quantifications of the western blots were carried out from membranes of freshly prepared lysates. Four sets of independent cell seedings and stimulations were prepared. The confocal microsopy experiments were repeated on three to four different days with independent transfections and multiple independent ligand stimulations per experimental day. Of these, at least 30 individual images per condition were analysed. Again based on our experience this number is large enough to identify individual variations in cell transfections. |
| Data exclusions | No datasets or individual measurements were excluded, all source data are additionally provided with this paper. |
| Replication | Experiments were repeated as described above and in the methods part or figure legends. All experiments shown could be reproduced as described. |
| Randomization | This study is entirely based on in cellulo experiments. Thus, no allocation into experimental groups was needed which could be randomized. |
| Blinding | Investigators were not blinded during data collection or analysis. This was not possible since, transfection of the cells was conducted by the same investigators. |

# Reporting for specific materials, systems and methods

We require information from authors about some types of materials, experimental systems and methods used in many studies. Here, indicate whether each material, system or method listed is relevant to your study. If you are not sure if a list item applies to your research, read the appropriate section before selecting a response.

## Materials & experimental systems

| n/a | Involved in the study | |
|---|---|---|
| ☐ | ☒ | Antibodies |
| ☐ | ☒ | Eukaryotic cell lines |
| ☒ | ☐ | Palaeontology and archaeology |
| ☒ | ☐ | Animals and other organisms |
| ☒ | ☐ | Human research participants |
| ☒ | ☐ | Clinical data |
| ☒ | ☐ | Dual use research of concern |

## Methods

| n/a | Involved in the study | |
|---|---|---|
| ☒ | ☐ | ChIP-seq |
| ☒ | ☐ | Flow cytometry |
| ☒ | ☐ | MRI-based neuroimaging |

## Antibodies

| | |
|---|---|
| Antibodies used | Western Blot secondary antibodies were purchased from SeraCare, goat anti-rabbit Catalog No: 5220-0336 and goat anti-mouse Catalog No: 5220-0341, both used 1:10,000<br>Vinculin: Biozol, BZL 03106; 1:1,000<br>pERK: phospho-p44/42, Cell signaling technology #9106; 1:1000, clone E10<br>total ERK: p44/42, Cell signaling technology #9107; 1:1000, clone 3A7 |
| Validation | total ERK<br>https://www.cellsignal.com/products/primary-antibodies/p44-42-mapk-erk1-2-3a7-mouse-mab/9107 |

pERK
https://www.cellsignal.com/products/primary-antibodies/phospho-p44-42-mapk-erk1-2-thr202-tyr204-e10-mouse-mab/9106

additional validation can be found via:
vinculin
https://doi.org/10.3390/ijms23031195
https://doi.org/10.1038/s41467-022-28152-8

## Eukaryotic cell lines

Policy information about cell lines

| | |
|---|---|
| Cell line source(s) | HEK293 cells were originally obtained from DSMZ Germany (ACC305). The HEK293 cell knockout derivatives were either created in-house (ΔQ-GRK cells, https://doi.org/10.1038/s41467-022-28152-8) or provided by co-authors (β-arrestin1/2 double knockout cells, doi:10.1126/scisignal.aal3395) |
| Authentication | Cells were not further authenticated by the authors. For GRK knockout cells, see https://doi.org/10.1038/s41467-022-28152-8 |
| Mycoplasma contamination | All used cell lines were regularly checked for mycoplasma infection by using the LONZA MycoAlert mycoplasma detection kit (LT07-318) and were found to be negative. |
| Commonly misidentified lines (See ICLAC register) | We did not use commonly misidentified cell lines. |
